# Effects of tegaserod on bile composition and hepatic secretion in Richardson ground squirrels on an enriched cholesterol diet

**DOI:** 10.1186/1476-511X-5-15

**Published:** 2006-06-22

**Authors:** Ronald Mathison, Eldon Shaffer, Hans-Juergen Pfannkuche, David Earnest

**Affiliations:** 1 Department of Physiology and Biophysics, Faculty of Medicine, University of Calgary, Calgary, AB, 2N 4N1, Canada; 2 Department of Medicine, Faculty of Medicine, University of Calgary, Calgary, AB, T2N 4N1, Canada; 3 Novartis Pharma AG, Basel, Switzerland; 4 Novartis Pharmaceuticals Corp., New Hanover, NJ, US

## Abstract

**Background:**

Tegaserod is effective in treating IBS patients with constipation, and does not alter gallbladder motility in healthy individuals or in patients with IBS. However, it is not known if tegaserod affects the biliary tract in gallstone disease, so to this end the effects of tegaserod on bile composition and hepatic secretion of Richardson ground squirrels maintained on an enriched cholesterol diet were examined.

**Results:**

Animals were fed either a control (0.03%) or enriched (1%) cholesterol diet for 28 days, and treated s.c. with tegaserod (0.1 mg/kg BID) or vehicle. Bile flow, bile acid, phospholipids and cholesterol secretion were measured with standard methods. Tegaserod treatment or enriched cholesterol diet, alone or combination, did not alter body or liver weights. The enriched cholesterol diet increased cholesterol saturation index (CSI), cholesterol concentrations in gallbladder and hepatic duct bile by ~50% and decreased bile acids in gallbladder bile by 17%. Tegaserod treatment reversed these cholesterol-induced changes. None of the treatments, drug or diet, altered fasting gallbladder volume, bile flow and bile salts or phospholipid secretion in normal diet and cholesterol-fed animals. However, tegaserod treatment prevented the decreases in bile acid pool size and cycling frequency caused by the enriched cholesterol diet, consequent to re-establishing normal bile acid to concentrations in the gall bladder. Tegaserod had no effect on these parameters with normal diet animals.

**Conclusion:**

Tegaserod treatment results in increased enterohepatic cycling and lowers cholesterol saturation in the bile of cholesterol-fed animals. These effects would decrease conditions favorable to cholesterol gallstone formation.

## Background

Irritable bowel syndrome (IBS) is a poorly understood symptom complex with abdominal pain and altered bowel function. Its basis likely involves visceral hypersensitivity and altered intestinal motility, perhaps mediated by the serotonin (5-HT) receptors [1]. IBS is not merely a motility disorder confined to the colon, but may involve the entire gut, urinary tract and gallbladder. As to the involvement of gallbladder in IBS, the reports have been conflicting. A small percentage of patients with IBS may have gallbladder disease[2], and some studies have shown normal[3,4], others enhanced[5], or impaired [6]gallbladder emptying in IBS. Despite these contradictions there is a higher rate of cholecystectomy in patients with IBS than that predicted in the general population[5,7].

One of the drugs being used for IBS that influences gut motility is tegaserod. Tegaserod, initially identified as a 5-HT_4 _partial agonist[8] and subsequently shown to antagonize 5-HT_2B _receptors[9] increases intestinal transit[10] and affects visceral afferent function by blunting the somatic reflex to colonic distension[11]. Consequent to these effects on colonic motor and sensory function, tegaserod is indicated for the treatment of irritable bowel syndrome with constipation in women (reviewed in[12]). Although functional 5-HT_4 _receptors have not been demonstrated in the gallbladder or biliary tract, cisapride, a 5-HT_4_-agonist/5-HT_3_-antagonist, has been variably reported to increase, decrease, and have no effect on gallbladder volume in human subjects[13-15]. Our previous work[16] with cisapride, in the Richardson ground squirrel model of cholesterol gallstone formation, revealed that this prokinetic agent reversed the defect in gallbladder contractility, enhanced bile salt secretion and so lowered cholesterol saturation. This increased hepatic bile salt secretion despite a reduction in the bile salt pool size, lowered the cholesterol saturation of bile, and reduced crystal nucleation and stone formation. Studies on the effect of tegaserod on hepatobiliary function are limited, although recently this drug was shown to have no significant effect on gallbladder contractility or the diameter of the common bile and hepatic ducts during both the interdigestive (fasting) and the digestive (postprandial) periods in healthy female subjects and female patients with IBS[17].

Thus, the objective of this study was to assess the effects of tegaserod on the enterohepatic circulation in a well-established animal model of gallbladder disease, the Richardson ground squirrel.

## Results

### Animal health

All animals remained in good health over the duration of the study, and tolerated the diets and daily interventions. There were no significant changes in initial and final body weights or the weights of the liver between the four groups of treatment animals (Table 1).

**Table 1:** Animal Characteristics

Normal Diet			1% Cholesterol Diet	
	Vehicle	Tegaserod	Vehicle	Tegaserod

Initial Body Weight (g)	558 ± 15	541 ± 12	508 ± 24	523 ± 27
Final Body Weight (g)	537 ± 13	535 ± 26	517 ± 24	503 ± 23
Liver Weight (g)	12.8 ± 1.1	12.5 ± 0.9	12.1 ± 0.8	11.8 ± 0.4
Gallbladder Weight (mg)	23.6 ± 1.4	24.8 ± 2.2	22.6 ± 1.5	23.6 ± 1.2

### Gallbladder lipid composition (Figure 1)

**Figure 1:**
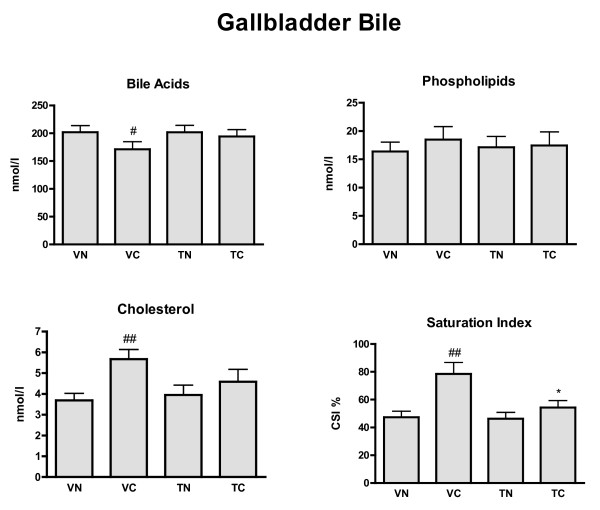
**Biliary lipid concentrations and cholesterol saturation index for gallbladder bile**. Bile acids, phospholipids, cholesterol concentrations (nmol/l) and cholesterol saturation index (CSI %) were measured in four groups of animals: VN = vehicle + normal diet (n = 17), VC = vehicle + cholesterol diet (n = 15), TN = tegaserod + normal diet (n = 15), and TC = tegaserod + cholesterol diet (n = 17). Significance (P < 0.05): * less than VC; # less than VN; ## greater than VN.

After an overnight fast cholesterol-fed, vehicle-treated (VC) animals had a higher concentration of cholesterol in gallbladder bile relative to normal diet animals (VN). In addition, gallbladder bile of the VC animals had a significantly lower concentration of bile acids relative to VN. These changes translated into a higher CSI in VC gallbladder bile than with VN. Tegaserod treatment did not modify the composition of gallbladder bile in animals on a normal diet (TN), but prevented the TC-induced changes in bile acids and cholesterol so that CSI was normalized to the values measured in VN.

### Hepatic duct bile lipid composition (Figure 2)

**Figure 2:**
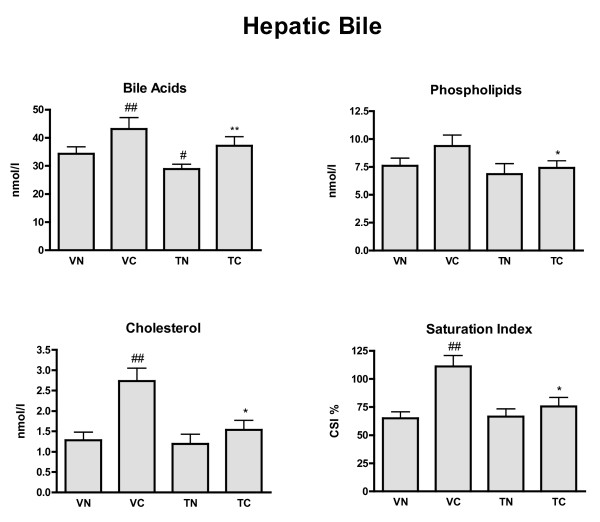
**Biliary lipid concentrations and cholesterol saturation index for hepatic duct bile**. Bile acids, phospholipids, cholesterol concentrations (nmol/l) and cholesterol saturation index (CSI %) were measured in four groups of animals: VN = vehicle + normal diet (n = 15), VC = vehicle + cholesterol diet (n = 15), TN = tegaserod + normal diet (n = 16), and TC = tegaserod + cholesterol diet (n = 17). Significance (P < 0.05): * less than VC; ** greater than TN; # less than VN; ## greater than VN.

Relative to VN the concentrations of bile acids and cholesterol in hepatic duct bile of VC were increased, although phospholipids were not affected. CSI was thus increased in VC. Tegaserod (TC) reversed these increased. With TN a small decrease in hepatic bile acid concentration occurred, but phospholipids and cholesterol concentrations remained unchanged relative to VN.

### Biliary lipid secretion rates, pool size and cycling time (Figure 3)

**Figure 3:**
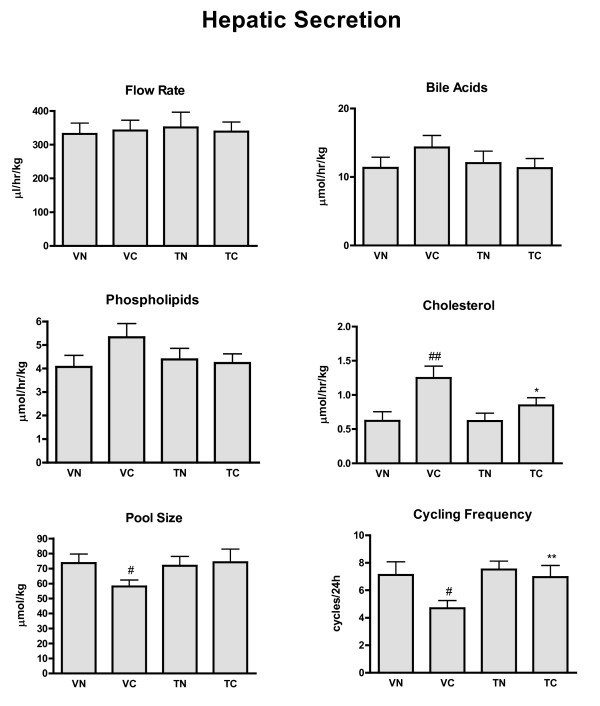
**Hepatic flow rates, pool size and cycling frequency**. Hepatic bile flow rate (μl/hr/kg), flow rates (μmol/hr/kg) for bile acids, phospholipids, cholesterol, pool size (μmol/kg) and cycling frequency (cycles/24 h) were measured in four groups of animals: VN = vehicle + normal diet (n = 14), VC = vehicle + cholesterol diet (n = 11), TN = tegaserod + normal diet (n = 16), and TC = tegaserod + cholesterol diet (n = 16). Significance (P < 0.05): * less than VC; ** greater than VC; # less than VN; ## greater than VN.

Hepatic bile flow and the secretion rates for bile acids and phospholipids were the same for the four treatment groups. The ~100% increase in cholesterol secretion that occurred with VC was reversed in TC. A reduction of bile acid pool size occurred in VC, due to the lower concentrations of bile acids in the gallbladder, and thus the cycling rate of the bile acid pool was decreased. Tegaserod treatment re-established pool size and normal cycling rate in TC, but did not modify cycling frequency in TN.

## Discussion

The Richardson ground squirrel fed a cholesterol-enriched diet serves as a model of gallstone disease[16,18-20], as this animal model exhibits impaired gallbladder contractility and increased cholesterol saturation index and cholesterol secretion, and decreased secretion of bile acids; the classical conditions favoring the formation of gallstones in obese humans[21,22]. The results of the current study concord with these previous observations in that the cholesterol-enriched diet increased CSI, decreased gallbladder bile acids concentrations, and enhanced cholesterol secretion and accumulation in gallbladder bile (Figure 1), but did not influence bile flow[23,24]. These conditions would favor lithogenesis. Although the previously reported[19,20] decrease bile acid secretion with VC animals was not observed in the present study, the concentrations of bile acids in the gallbladder were significantly reduced (Figure 1), and overall an increase in cholesterol concentrations in both gallbladder and hepatic bile, and a decrease in gallbladder bile acids, contributed predominately to an increase in CSI characteristic of an enriched cholesterol diet[16,18-20].

Tegaserod, a prokinetic agent[25] is associated with improved bile composition as reflected in an improved enterohepatic circulation (Figure 3). This result contrasts with the improved bile acid secretion and negligible effects on cholesterol secretion seen with another prokinetic agent cisapride[16], but concords with reduced cholesterol concentrations and CSI in patients with gallstone disease treated with cisapride[26]. The cholesterol lowering effects of cisapride has been attributed to enhanced bile salt secretion[16], but since bile acid secretion was not increased in TC animals, an alternative mechanism must be responsible for the cholesterol-lowering effects of tegaserod. Two possible inter-related mechanisms may explain this corrective action of tegaserod.

Ground squirrels fed a VC diet had a prolonged migrating myoelectric complex cycle (MMC) period[20], increased cholinergic contractions of intestinal smooth muscle[27] and impaired enterohepatic cycling[20] (Figure 3). The improvement of enterohepatic circulation could occur in TC animals consequent to increased gastrointestinal transit[28], with resulting normalization of the cholesterol composition of hepatic duct bile (Figure 2). The increased cholinergic contractions in VC animals, which could disrupt MMCs[29], may arise from cholesterol metabolites blocking inhibitory neuronal muscarinic receptors.[27,30]

## Conclusion

Tegaserod, a prokinetic agent used to treat IBS in women, increased enterohepatic cycling and lowers cholesterol saturation in the bile of cholesterol-fed animals. Thus, tegaserod treatment may decrease conditions favorable to cholesterol gallstone formation.

## Methods

### Experimental animals

The University of Calgary Animal Care Committee approved the researchprotocol, which conforms to the guidelines of the Canadian Council on Animal Care. Eighty Male Richardson ground squirrels (*Spermophilus richardsoni*) were trapped wild near Calgary, and acclimatized for a minimum period of four weeks by caging them individually in thermoregulated rooms on a 12 h/12 h day/night light cycle with free access to a standard rat chow diet. After acclimatization the animals were fed, under the same holding conditions for four weeks, either a normal (control) diet with trace cholesterol content (0.027%, Dyets Inc., Bethlehem, PA) or an enriched cholesterol diet (Dyets Inc.) comprising an identical chow, enriched with 1% cholesterol by weight. During this diet period the animals were treated subcutaneously, twice daily, with either vehicle or 0.1 mg/kg of tegaserod. Tegaserod was dissolved in vehicle (100% 1-methyl-2-pyrrolidinone) immediately before use, and then diluted into 0.9% saline such that the final concentration of 1-methyl-2-pyrrolidinone was 2.7%. The vehicle-treated animals received 0.9% saline containing 2.7% 1-methyl-2-pyrrolidinone Sigma-Aldrich, St. Louis, MO). The animals were randomly divided into four groups (20 in each) that received the following combination of diet and drug: (1) vehicle + normal diet (VN), (2) vehicle + cholesterol diet (VC), (3) tegaserod + normal diet (TN), and (4) tegaserod + cholesterol diet (TC).

### Hepatic secretion and bile salt kinetics

Acute terminal experiments *in *vivo meanwhile directly measured bile output, biliary lipid secretion and bile composition, and determine the bile salt pool size by isotope dilution. Fourteen hours before the acute terminal studies each animal was lightly and briefly sedated with halothane (Ayerst Laboratories, Montreal, Canada) and 0.2 μCi of tritiated taurocholic acid, (PerkinElmer Life and Analytical Sciences, Inc., Boston, MA) was injected directly by cardiac puncture. Animals were ambulatory within 5 minutes.

After a 14-hour overnight fast, animals were anesthetized with isoflurane (BioMeda MTC, Cambridge, Ontario) and maintained on a low concentration (1.5 to 2%) isoflurane. At laparotomy the cystic duct was exposed and ligated, the gallbladder removed and the common bile duct cannulated with a polyethylene catheter (PE50). Hepatic bile was collected into tared tubes on an hourly basis for a total of 2 hours and frozen at -70°C for later analysis. During this period, 154 mmol/L NaC1 was infused continuously at 2.5 mL/h via a femoral venous cannula for fluid replacement. Terminally, the liver was wet weighed. Gallbladder bile was aspirated and the volume measured, before storage at -70°C for later analysis.

### Assay of bile lipids

Bile acids were analyzed using the 3-α hydroxysteroid dehydrogenase colorimetric assay[31] as developed into Total Bile Acid Test Kit (enzyme cycling) by Diazyme (San Diego, CA). This assay has linear range of 1–180 μM. Total cholesterol (cholesterol and cholesteryl ester) was measured using a method based on the Liebermann-Burchard reaction[32], and as developed into a Cholesterol/Cholesteryl Ester Quantitation Kit of Biovision (Mountain View, CA). Phospholipids were measured as total biliary phosphate after hydrolysis with sulphuric acid, using a modification (Chen et al, 1965) of the colorimetric assay of Fiske & Subbarow[33]. The results are expressed as micromole of total bile acids per liter.

### Calculations and statistical analysis

The relative lipid biliary composition, calculated after the method of[22], was expressed as percentage molar composition. The CSI of each sample was calculated in native bile using Carey's critical tables[34,35] for cholesterol solubility limits based on total lipid concentration. Biliary secretion rate, calculated by multiplying the bile flow (microliters per hour per kilogram body weight) with the solute concentration (millimolar) in fraction of solute collected over a 1 h period, was expressed as nmol·h^-1^·kg body weight^-1^. The bile acid pool size was calculated using a modified isotope dilution technique[16,36]. Cycling time, defined as the time it takes bile acids to circulate once in the enterohepatic circulation, was calculated by dividing the bile acid pool size (micromoles·kg body weight^-1^) by the bile acid secretion rate (micromoles·h^-1^·kg body weight^-1^).

The results are presented as the mean ± SEM. The Z-test was used to identify and exclude outliers that were more than 3 SD from the mean. The statistical functions associated with Excel (Microsoft Office XP, Redmond, WA) were used. Comparisons between two groups were made using the unpaired Student's t-test, and where appropriate analysis of variance was applied. Statistical values reaching probabilities of p < 0.05 were considered significant.

## Abbreviations

CSI – cholesterol saturation index; IBS – irritable bowel syndrome; TC – tegaserod + cholesterol diet; TN – tegaserod + normal diet; VC – vehicle + cholesterol diet; VN – vehicle + normal diet

## Competing interests

This work was supported by Novartis Pharma AG, Basel, Switzerland. The author(s) declare that they have no competing interests.

## Authors' contributions

RM carried out the experimental studies and drafted the manuscript. HJF provided tegaserod and defined treatment schedules and dosing. ES, HJF and DE conceived of the study, and participated in its design and coordination and helped to draft the manuscript. All authors read and approved the final manuscript.
